# exoRBase 3.0: an updated resource for extracellular RNAs from human biofluids

**DOI:** 10.1093/nar/gkaf1004

**Published:** 2025-10-16

**Authors:** Wenqian Yu, Jia Hu, Peng Lin, Jingjing Zhao, Hena Zhang, Yongxiang Wang, Yaoming Su, Yan Li, Zhuting Fang, Zhaohui Huang, Zhiqiang Meng, Shenglin Huang

**Affiliations:** Department of Integrative Oncology, Fudan University Shanghai Cancer Center, and Shanghai Key Laboratory of Medical Epigenetics, Institutes of Biomedical Sciences, Fudan University, Shanghai 200032, China; Department of Oncology, Shanghai Medical College, Fudan University, Shanghai 200032, China; Department of Integrative Oncology, Fudan University Shanghai Cancer Center, and Shanghai Key Laboratory of Medical Epigenetics, Institutes of Biomedical Sciences, Fudan University, Shanghai 200032, China; Department of Oncology, Shanghai Medical College, Fudan University, Shanghai 200032, China; Department of Integrative Oncology, Fudan University Shanghai Cancer Center, and Shanghai Key Laboratory of Medical Epigenetics, Institutes of Biomedical Sciences, Fudan University, Shanghai 200032, China; Department of Oncology, Shanghai Medical College, Fudan University, Shanghai 200032, China; Department of Integrative Oncology, Fudan University Shanghai Cancer Center, and Shanghai Key Laboratory of Medical Epigenetics, Institutes of Biomedical Sciences, Fudan University, Shanghai 200032, China; Department of Oncology, Shanghai Medical College, Fudan University, Shanghai 200032, China; Department of Integrative Oncology, Fudan University Shanghai Cancer Center, and Shanghai Key Laboratory of Medical Epigenetics, Institutes of Biomedical Sciences, Fudan University, Shanghai 200032, China; Department of Oncology, Shanghai Medical College, Fudan University, Shanghai 200032, China; Department of Integrative Oncology, Fudan University Shanghai Cancer Center, and Shanghai Key Laboratory of Medical Epigenetics, Institutes of Biomedical Sciences, Fudan University, Shanghai 200032, China; Department of Oncology, Shanghai Medical College, Fudan University, Shanghai 200032, China; Department of Integrative Oncology, Fudan University Shanghai Cancer Center, and Shanghai Key Laboratory of Medical Epigenetics, Institutes of Biomedical Sciences, Fudan University, Shanghai 200032, China; Department of Oncology, Shanghai Medical College, Fudan University, Shanghai 200032, China; Department of Integrative Oncology, Fudan University Shanghai Cancer Center, and Shanghai Key Laboratory of Medical Epigenetics, Institutes of Biomedical Sciences, Fudan University, Shanghai 200032, China; Department of Oncology, Shanghai Medical College, Fudan University, Shanghai 200032, China; Department of Oncology and Vascular Interventional Therapy, Clinical Oncology School of Fujian Medical University, Fujian Cancer Hospital (Fujian Branch of Fudan University Shanghai Cancer Center), Fuzhou 350003, China; Wuxi Cancer Institute, Affiliated Hospital of Jiangnan University, Wuxi 214062, China; Department of Integrative Oncology, Fudan University Shanghai Cancer Center, and Shanghai Key Laboratory of Medical Epigenetics, Institutes of Biomedical Sciences, Fudan University, Shanghai 200032, China; Department of Oncology, Shanghai Medical College, Fudan University, Shanghai 200032, China; Department of Integrative Oncology, Fudan University Shanghai Cancer Center, and Shanghai Key Laboratory of Medical Epigenetics, Institutes of Biomedical Sciences, Fudan University, Shanghai 200032, China; Department of Oncology, Shanghai Medical College, Fudan University, Shanghai 200032, China; State Key Laboratory of Genetics and Development of Complex Phenotypes, Fudan University, Shanghai 200433, China

## Abstract

Extracellular RNAs (exRNAs) in human biofluids have emerged as promising liquid biopsy biomarkers for noninvasive disease diagnosis and monitoring. Here, we present exoRBase 3.0 (http://www.exorbase.org), a significantly expanded database that systematically integrates and compares extracellular long RNAs (exLRs) derived from both extracellular vesicles and particles (EVPs) and cell-free RNA fractions. The database encompasses 19 927 messenger RNAs, 15 961 long non-coding RNAs, and 126 816 circular RNAs from 2913 samples across blood, urine, cerebrospinal fluid, and bile. A key advancement is the first large-scale comparative analysis platform that distinguishes between EVP-associated and cell-free exLRs, enabling identification of context-specific biomarkers and optimization of liquid biopsy strategies. Enhanced with interactive visualizations and expanded pathway analysis covering 12 981 gene sets, exoRBase 3.0 facilitates comprehensive comparative analysis of expression profiles, functional pathways, and tissue/cell origins across different sample types and disease states. This resource addresses the critical need for understanding how RNA biomarker performance varies between EVP and cell-free contexts, ultimately advancing precision medicine through improved liquid biopsy approaches for cancer diagnosis, disease monitoring, and therapeutic applications.

## Graphical Abstract

**Graphical Abstract:**
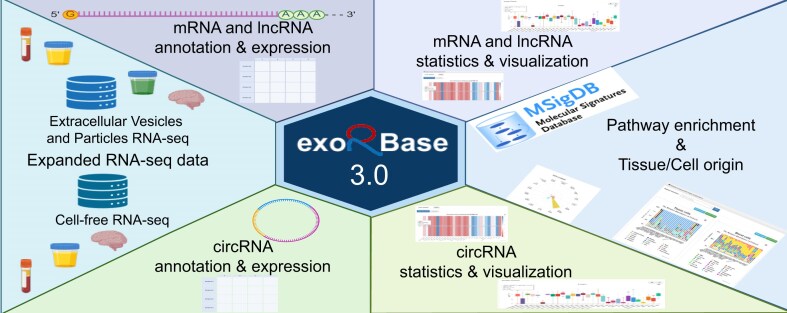

## Introduction

Extracellular RNAs (exRNAs) have emerged as key mediators of intercellular communication and hold great promise as biomarkers for liquid biopsy due to their remarkable stability in biofluids and disease-specific expression patterns [1, 2]. These RNA species—including messenger RNAs (mRNAs), long noncoding RNAs (lncRNAs), and circular RNAs (circRNAs)—can be released into the extracellular space via various mechanisms, such as encapsulation within extracellular vesicles (EVs), association with RNA-binding proteins or lipoproteins, or circulation as free ribonucleoprotein complexes [2]. Collectively referred to as extracellular long RNAs (exLRs), these molecules reflect the molecular state of their cells of origin and have been increasingly recognized for their potential in noninvasive diagnostics, therapeutic monitoring, and elucidating disease mechanisms [3, 4].

To support the growing interest in exLRs, we previously developed exoRBase, a dedicated database for the storage, annotation, and exploration of exLRs. The initial release, exoRBase 1.0 (2017), provided the first comprehensive resource for exosome-derived exLRs from human plasma [5]. Building on this foundation, exoRBase 2.0 significantly expanded the landscape of EV-associated exLRs, incorporating 19 643 mRNAs, 15 645 lncRNAs, and 79 084 circRNAs across nearly 1000 samples from plasma, urine, cerebrospinal fluid (CSF), and bile [6]. This version also introduced tools for functional enrichment analysis and tissue-of-origin inference [7, 8]. However, exoRBase 2.0 focused exclusively on EV-derived exRNAs, overlooking cell-free RNAs (cfRNAs) that circulate independently of vesicular structures and are increasingly valued for their diagnostic utility, particularly in prenatal screening and early cancer detection [9–11].

Recent advances have revealed a broader and more heterogeneous repertoire of exRNA carriers beyond classical EVs. Non-membranous nanoparticles such as exomeres and supermeres have been identified as novel vehicles for exRNA transport, expanding the definition of RNA carriers to encompass both vesicular and non-vesicular entities [12–14]. These structures are now collectively referred to as extracellular vesicles and particles (EVPs), reflecting their compositional and functional diversity [15]. In parallel, cfRNAs have gained recognition for their distinct biophysical properties and clinical relevance. Despite their shared extracellular origin, EVP- and cfRNA-associated exLRs exhibit divergent profiles and diagnostic potentials, underscoring the need for an integrated resource that enables comprehensive analysis and cross-comparison of these complementary RNA populations.

Here, we present exoRBase 3.0, a major update that substantially extends the scope and utility of the database by integrating exLR profiles derived from both EVP and cfRNA fractions. This release incorporates data from 2913 human biofluid samples—a three-fold increase over version 2.0—and expands transcript coverage to include 19 927 mRNAs, 15 961 lncRNAs, and 126 816 circRNAs. Notably, exoRBase 3.0 enables the systematic comparison of EVP- and cfRNA-derived exLRs, providing insights into their complementary biological roles and biomarker capacities. Functional annotation is supported by single-sample gene set enrichment analysis (ssGSEA) utilizing updated pathways from the latest version of MSigDB (Molecular Signatures Database) [16–18]. In addition, the platform incorporates cellular deconvolution for 16 tissue types and 23 immune cell populations based on the EV-origin method. By bridging these previously siloed datasets, exoRBase 3.0 offers a unified, scalable, and user-friendly platform to advance exRNA-based research and accelerate translational applications in precision medicine.

## Materials and methods

### Integration of available exLR profiling

We collected a comprehensive set of 2319 RNA-seq datasets of EVPs derived from human blood, urine, CSF, and bile, as well as 594 RNA-seq datasets of cfRNA from human blood, urine, and CSF. All datasets were integrated into the exoRBase 3.0 database (Table 1). Both EVP and cfRNA blood samples cover a variety of biological conditions, including healthy individuals, gastric cancer (GC), colorectal cancer (CRC), hepatocellular carcinoma (HCC), and non-small cell lung cancer (NSCLC). In addition, EVP blood samples also encompass benign conditions and a range of other diseases, such as breast cancer (BRCA), glioblastoma (GBM), kidney renal clear cell carcinoma (KIRC), melanoma (MEL), malignant lymphoma (ML), ovarian cancer (OV), pancreatic ductal adenocarcinoma (PDAC), and small cell lung cancer (SCLC). For cfRNA blood samples, additional disease states are included, such as esophageal carcinoma (ESCA), multiple myeloma (MM), monoclonal gammopathy of undetermined significance (MGUS), chronic hepatitis B (CHB), and liver cirrhosis. Detailed information on the source of each dataset can be found on the exoRBase 3.0 website (http://www.exorbase.org/statistics.html). To annotate the possible tissue origins of exLRs, gene expression profiles (TPM values) were also downloaded from the GTEx project (RNA-seq analysis V8, GENCODE version 26), covering 30 human tissues [19].

**Table 1.** Expanded data in exoRBase 3.0 compared with exoRBase 2.0 and exoRBase 1.0

Type	Cohort	exoRBase 1.0	exoRBase 2.0	exoRBase 3.0
EVPs in urine	EVPs in urine	–	16	125
EVPs in CSF	EVPs in CSF	–	5	5
EVPs in bile	EVPs in bile	–	17	21
EVPs in blood	Healthy	32	118	244
	Benign	–	130	372
	BRCA	2	140	242
	CRC	12	35	259
	GBM	–	13	13
	GC	–	9	15
	HCC	21	112	305
	KIRC	–	15	15
	MEL	–	21	51
	ML	–	28	44
	OV	–	30	58
	PDAC	14	164	365
	SCLC	–	36	75
	NSCLC	–	–	110
Urine (cfRNA)		–	–	30
CSF (cfRNA)		–	–	4
Blood (cfRNA)	Healthy	–	–	175
	CHB	–	–	20
	CRC	–	–	95
	ESCA	–	–	31
	GC	–	–	73
	HCC	–	–	104
	Liver cirrhosis	–	–	4
	NSCLC	–	–	35
	MGUS	–	–	9
	MM	–	–	14
Target	mRNA	18 333	19 643	19 927
	lncRNA	15 501	15 645	15 961
	circRNA	58 330	79 084	126 816
	Pathway	–	11 536	12 981
	Tissue/cell origin	–	39	39

Cohorts labeled with “EVPs” represent samples for which extracellular vesicles and particles were isolated and sequenced. Cohorts without the “EVPs” label represent cfRNA sequencing samples from the respective biofluid. CSF, cerebrospinal fluid; BRCA, breast cancer; CHB, chronic hepatitis B; CRC, colorectal cancer; ESCA, esophageal cancer; GBM, glioblastoma multiforme; GC, gastric cancer; HCC, hepatocellular carcinoma; KIRC, kidney cancer; MEL, melanoma; ML, malignant lymphoma; MM, multiple myeloma; MGUS, monoclonal gammopathy of undetermined significance; OV, ovarian cancer; PDAC, pancreatic ductal adenocarcinoma; SCLC, small cell lung cancer; NSCLC, non-small cell lung cancer.

### Identification, annotation, and quantification of exLRs with an improved pipeline

**Figure 1.**
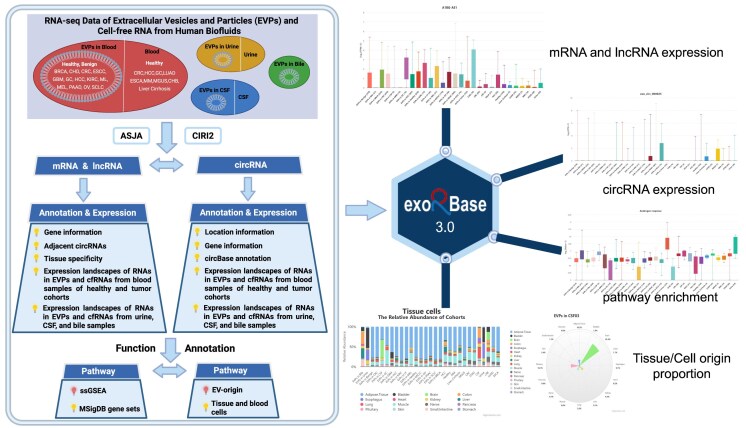
Overview of the core content and framework of exoRBase 3.0. ExoRBase 3.0 integrates RNA-seq data from EVPs isolated from human blood, urine, CSF, and bile samples, as well as from cfRNA samples obtained from blood, urine, and CSF. exLRs—including mRNAs, lncRNAs, and circRNAs—were identified, annotated, and quantified using the Assembling Splice Junctions Analysis (ASJA) and CircRNA Identifier (CIRI2) bioinformatic tools. To interpret exLR expression profiles, pathway enrichment analysis was performed on MSigDB gene sets using the ssGSEA method. The EV-origin algorithm was applied to estimate the proportions of tissue and blood cell sources contributing to exLRs. ExoRBase 3.0 provides visualization and comparative analysis of exLR expression patterns, pathway enrichment levels, and the origins of exLRs.

The data processing and analysis pipeline for exLR identification, annotation, and quantification followed the established procedures in exoRBase 2.0 [6] and continued to employ tools such as ASJA [20] and CIRI2 [21] for alternative splicing and circRNA analysis, respectively, with the primary update being the use of STAR version 2.7.8a for genome alignment instead of STAR version 2.7.1a (Fig. 1). The calculation of tissue specificity scores was performed in the same manner as in exoRBase 2.0 [6]. CircRNA annotation information from circBase was integrated as previously described in exoRBase 1.0 [5].

The definition of exLR sample types was updated in exoRBase 3.0 to reflect the increased diversity of sample categories. Specifically, samples were categorized based on whether exLRs were detected from EVPs or directly from the cfRNA fraction of biofluids. This classification resulted in distinct sample types such as “EVPs in Blood” for exLRs detected from EVPs isolated from blood, “Blood” for exLRs detected from cfRNA in blood, and analogous categories for other biofluids including “EVPs in Urine,” “Urine,” “EVPs in CSF,” “CSF,” and “EVPs in Bile.” The sample type annotation of each exLR was determined by its detection frequency across different sample types. For blood-related categories, an exLR required detection in at least three samples to receive the corresponding annotation: either “EVPs in Blood exLR” for those detected in EVPs in blood samples or “Blood exLR” for those detected in blood samples. For other biofluids, an exLR detected in at least one sample would be annotated according to its respective sample type, yielding classifications such as EVPs in Urine exLR, EVPs in CSF exLR, EVPs in Bile exLR, Urine exLR, or CSF exLR.

Given the integration of datasets across multiple studies and biofluids, potential batch effects were carefully considered. To mitigate technical variability, a standardized preprocessing and normalization pipeline was applied. Raw reads first underwent adaptor trimming using fastp [22], followed by quality filtering at Q30, and alignment to the GRCh38 reference genome with STAR (v2.7.8a). Transcript filtering retained only those with TPM > 0.5 in at least 1% of samples, and TPM values were then log2-transformed (log2[TPM + 1]) for downstream analyses. These steps provide a consistent basis for integrative analyses while reducing study-specific biases.

### Pathway enrichment analysis with expanded MSigDB collections

In exoRBase 3.0, pathway enrichment analysis was conducted using an expanded set of gene sets from MSigDB (version v2025.1.Hs; https://www.gsea-msigdb.org/gsea/msigdb/) [17], comprising 12 981 annotated pathways. This collection includes 50 hallmark gene sets [18], 292 BIOCARTA [23], 186 KEGG [24], 1787 REACTOME [25], 7583 biological process, 1852 molecular function, and 1042 cellular component gene sets from Gene Ontology [26, 27], as well as 189 oncogenic signature gene sets. Enrichment scores for each pathway were calculated per sample using the ssGSEA method implemented in the GSVA R package [28]. Apart from the expanded pathway collections, the analytical procedure was consistent with that of exoRBase 2.0.

### Tissue and cellular origin estimation with interactive visualization

Tissue and cellular origin estimation was performed using the EV-origin algorithm [8], as in exoRBase 2.0, to quantify the contributions of 16 human tissues and 23 blood cell types based on exLR expression profiles. In exoRBase 3.0, origin analysis results are visualized online using Highcharts, including cumulative percentage charts and rose polar diagrams. These interactive web-based plots allow users to select or hide specific tissue or cell type components and to access detailed statistics by hovering over individual elements.

### Advanced statistical analysis and interactive visualization of exLR targets: homogeneity and heterogeneity insights

In exoRBase 3.0, exLRs are first stratified by their biogenesis, distinguishing those detected in EVPs from those identified as cfRNAs in various biofluids. Specifically, expression patterns, pathway enrichment scores, and relative abundance of tissue/cell origins are calculated separately for EVPs in urine, CSF, bile, and for healthy, benign, and tumor EVP samples from blood. Equivalent analyses are also performed for exLRs present as cfRNAs in urine, CSF, and in healthy, benign, and tumor blood samples.

For each category, expression frequencies, sample numbers, and mean expression values of exLRs are computed. Pathway enrichment scores and the relative abundance of tissue/cell origins are also calculated for all cohorts. Visualization includes line charts and heatmaps for mean expression values, as well as boxplots for comparing expression levels, pathway enrichment scores, and relative abundance of tissue/cell origins across different groups.

To identify differentially expressed or enriched targets, the Mann–Whitney *U-*test was used to separately perform differential analysis between disease or tumor cohorts and healthy controls based on both EVP and cfRNA sequencing data. For the EVP-sequenced healthy group, which contains 244 samples, differential analysis for cohorts with smaller sample sizes (e.g. BRCA, GC, GB, KIRC, MEL, ML, OV, SCLC, and NSCLC) is conducted by randomly selecting an equal number of healthy EVP samples to ensure balanced comparisons. For cohorts with larger sample sizes (e.g. Benign, CRC, HCC, and PDAC), the number of samples is matched to the healthy EVP group for consistency. For cfRNA sequencing data, differential analysis is performed by comparing each disease or tumor group to matched healthy cfRNA-sequenced controls, applying the same sample number adjustment strategy.

Long RNAs (including lncRNAs and mRNAs) and circRNAs are considered significant if they meet the criteria of |log_2_ fold change| > 1 and *P*-value < 0.05. Enrichment results from pathway analysis and the EV-origin algorithm are included if they meet the criterion of *P*-value < 0.05. This approach is used to ensure robust identification of differentially expressed or enriched targets while mitigating potential biases due to sample size imbalances. All statistical analyses are conducted in R (version 4.0.2).

In exoRBase 3.0, visualization of expression levels, pathway enrichment scores, and relative abundance of tissue/cell origins is performed using interactive, web-based Highcharts, replacing the static plots used in previous versions. Boxplots, heatmaps, and line charts can now be interactively explored online, with detailed statistics available through user interaction.

### Database construction

The exoRBase 3.0 website was developed using standard web technologies to ensure efficient data access and user interaction. The frontend interface was built with HTML and CSS for structure and styling, while JavaScript was used to implement interactive logic and client-side functionality. Node.js was employed for backend data processing, enabling rapid data retrieval and loading. For data visualization, Highcharts (version 5.0.12) was integrated to provide dynamic and interactive charts throughout the platform. The website is fully responsive and compatible with major browsers such as Google Chrome, Microsoft Edge, Mozilla Firefox, and Safari, allowing users to access and interact with the database seamlessly on both desktop and mobile devices.

## Updated database content and user interface

Overall, exoRBase 3.0 significantly expands upon previous versions by integrating exLRs data not only from EVPs but also from cell-free exLRs directly present in body fluids (Fig. 1). This update substantially increases the number of EVP-derived exLR datasets and introduces a new layer of data from circulating cell-free exLRs. A key feature of exoRBase 3.0 is the ability to systematically compare target expression frequencies, mean expression levels, and detection status between EVP-associated and cell-free exLRs. The database allows users to explore whether targets are consistently highly expressed, consistently lowly expressed, or differentially expressed depending on the source. Additionally, exoRBase 3.0 enables cross-source comparisons of pathway enrichment analyses to determine whether shared or distinct pathways are identified from different exLR sources, as well as comparisons of tissue/cell-of-origin estimations for each exLR source. Apart from these major advances, the analytical framework, including pathway enrichment analysis and EV-origin estimation, remained consistent with exoRBase 2.0. Another important improvement in exoRBase 3.0 is the transition from static to interactive and dynamic data visualizations, providing users with a more flexible and informative exploration experience. Together, these enhancements make exoRBase 3.0 a more comprehensive and versatile resource for the study of exLRs in human body fluids.

### Increased data coverage and new features

This updated database comprises high-quality exLR-seq data from EVPs isolated from blood, urine, CSF, and bile samples, collected from a total of 2319 individuals. Compared to the previous 2.0 version, the number of EVP samples from urine has increased from 16 samples to 125 samples, CSF samples remain at 5 samples, and bile samples have increased from 17 samples to 21 samples. The number of EVP samples from blood has also grown substantially in exoRBase 3.0, now including healthy participants (244 samples), patients with benign disease (372 samples), and individuals diagnosed with BRCA (242 samples), CRC (259 samples), GBM (13 samples), GC (15 samples), HCC (305 samples), KIRC (15 samples), MEL (51 samples), ML (44 samples), OV (58 samples), PDAC (365 samples), SCLC (75 samples), and NSCLC (110 samples). ExoRBase 3.0 also introduces exLR-seq data from cell-free exLRs directly isolated from body fluids, comprising samples from 594 individuals: urine (30 samples), CSF (4 samples), and blood (560 samples). The blood cfRNA samples are grouped into 10 cohorts: Healthy (175 samples), CHB (20 samples), CRC (95 samples), ESCA (31 samples), GC (73 samples), HCC (104 samples), liver cirrhosis (4 samples), NSCLC (35 samples), MGUS (9 samples), and MM (14 samples). Through comprehensive RNA-seq analysis, exoRBase 3.0 provides annotation and expression profiles for 19 927 mRNAs, 15 961 lncRNAs, and 126 816 circRNAs detected in both EVP-associated and cell-free fractions of human body fluids. Notably, this version also includes enrichment scores for 12 981 MSigDB pathways, as well as relative abundance for 39 tissue and cell origins for each sample, calculated based on exLR expression profiles. Table 1 summarizes the expanded datasets and new features incorporated into exoRBase 3.0.

### Extended comparison platform for EVPs and cfRNA

The basic functional interfaces of the new exoRBase 3.0 remain consistent with those of exoRBase 2.0, including the Browse, Search and Results, Origin, and Detail pages. For detailed descriptions of these interfaces, please refer to exoRBase 2.0 [6]. In the new version, additional exLR comparison platforms have been introduced, enabling direct comparison of exLRs detected in EVPs and those present in a cell-free state.

#### Comprehensive assessment of target detection status across EVPs and cell-free exLRs

**Figure 2.**
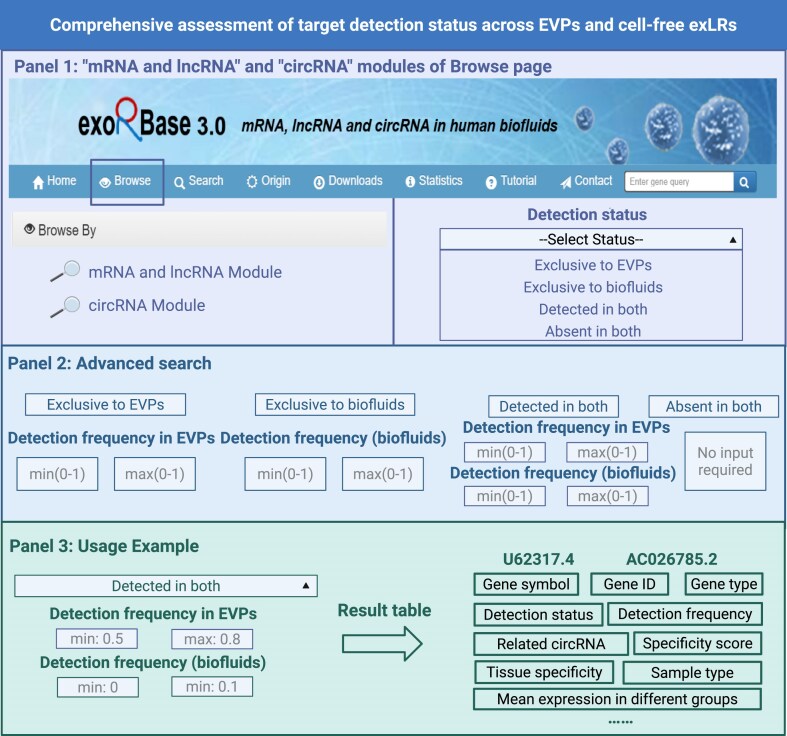
Flexible detection status filtering of exLR targets. Panel 1 describes the detection status selection interface in the Browse page for both mRNA and lncRNA module and circRNA module, displaying four selectable categories: Exclusive to EVPs, Exclusive to biofluids, Detected in both, and Absent in both. Panel 2 describes the advanced search interface that enables users to set specific detection frequency thresholds for EVPs and biofluids across different detection status categories. Panel 3 describes a usage example demonstrating the filtering process where “Detected in both” status is selected with detection frequency ranges of 0.5–0.8 for EVPs and 0–0.1 for biofluids, resulting in the identification of two lncRNA targets (U62317.4 and AC026785.2) with comprehensive target information displayed in the result table.

In exoRBase 3.0, a key feature is the ability to systematically assess and compare the detection status of exLR targets, including mRNAs, lncRNAs, and circRNAs across two distinct biological contexts: those encapsulated within EVPs and those freely circulating as cfRNAs in body fluids. This allows users to determine whether a specific target is exclusively detected in EVPs, exclusively in biofluid cfRNAs, detected in both, or absent in both. The “Detection status” option available in the Browse interface of the mRNA, lncRNA, and circRNA modules provides four selectable categories: Exclusive to EVPs, Exclusive to biofluids, Detected in both, and Absent in both (Fig. 2, Panel 1). By default, “Exclusive to EVPs” filters out targets with a detection frequency greater than zero in EVP sample groups and zero in cfRNA groups. “Exclusive to biofluids” selects targets with a detection frequency greater than zero in cfRNA groups and zero in EVP groups. “Detected in both” returns targets with detection frequency greater than zero in both sources, while “Absent in both” identifies targets undetected in both contexts.

To further refine analyses, an advanced search mode is provided (Fig. 2, Panel 2). This enables users to specify detection frequency thresholds for individual sample groups. For example, when “Exclusive to biofluids” is selected, users can define detection frequency in cfRNA groups. Similarly, “Exclusive to EVPs” allows for precise filtering based on detection frequency in EVP groups. For “Detected in both,” users can set distinct frequency ranges for both EVPs and cfRNAs. If “Absent in both” is chosen, advanced frequency options are not available, as both frequencies are zero by definition.

Figure 2, Panel 3 demonstrates the filtering functionality using the Browse interface for mRNAs and lncRNAs as an example. The search process for circRNAs follows the same approach. The left panel allows users to input filtering criteria, while the right panel displays the search results. For instance, when users are interested in target detected in both contexts but wish to identify those with preferential presence in EVPs over cfRNAs, they can select “Detected in both” status and set detection frequency ranges accordingly (e.g. 0.5–0.8 for EVPs and 0–0.1 for cfRNAs). In this example, the filtering identified two lncRNA targets: U62317.4 and AC026785.2. The search results provide detailed information for each target, including gene type, gene symbol, average expression levels across different sample groups, detection frequency, differential expression patterns, associated diseases, and other relevant characteristics.

This comprehensive detection status framework enables nuanced comparisons of target RNA presence between EVPs and cell-free states. It allows users to identify targets that are specifically present in EVPs or exclusively in the cell-free state and to select the most appropriate liquid biopsy approach for different disease contexts.

#### Comparative profiles of exLRs expression frequency and level in EVPs and cfRNAs

**Figure 3.**
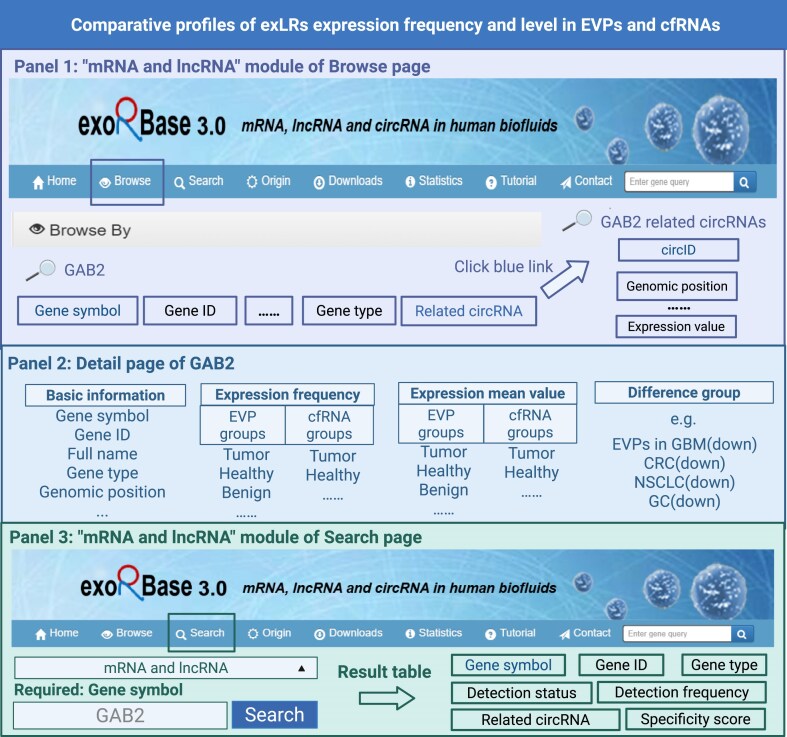
Comparative profiles of exLRs expression frequency and level in EVPs and cfRNAs. Panel 1 describes the “mRNA and lncRNA” module of the Browse page showing GAB2 gene search results with basic information fields and a blue “Related circRNA” link that leads to GAB2-related circRNAs information. Panel 2 describes the detail page of GAB2 displaying four major information categories. Panel 3 describes the “mRNA and lncRNA” module of the Search page where users can input “GAB2” to retrieve gene information displayed in the result table with comprehensive target details.

Users can explore exLRs of interest (including mRNAs, lncRNAs, and circRNAs) via the Browse and Search interfaces. Here, we use the mRNA and lncRNA interfaces as examples. For instance, if GAB2 is of interest, users can search for this gene in both the Browse and Search pages. In the Browse interface (Fig. 3, Panel 1), after searching, the basic information for the gene is displayed. Clicking the blue link under “gene symbol” navigates to the detail page for this gene. If there are associated circRNAs, the blue link under “Related circRNA” will show information for the corresponding circRNA; clicking the blue circID link leads to the circRNA’s detail page, which has a similar information composition as the mRNA and lncRNA detail pages (Fig. 3, Panel 2). In the mRNA and lncRNA sections of the Search interface, searching for “GAB2” and clicking the Search button displays the gene entry’s basic information (Fig. 3, Panel 3). Similarly, clicking the blue gene symbol link opens the detailed gene page, where comprehensive information is provided. The detailed gene page presents four major categories of information: (i) Basic information including gene symbol, gene ID, full name, gene type, and other relavant information; (ii) Expression frequency data subdivided into EVPs sample groups and cfRNAs sample groups; (iii) Average expression levels across different sample groups; and (iv) Differential expression analysis between various groups. For example, GAB2 shows significant downregulation in EVPs from GBM patients compared to EVPs from healthy individuals, while exhibiting significant upregulation in CRC cell-free body fluids compared to corresponding controls. Such comparative expression profiles across EVPs and cfRNAs enable researchers to identify compartment-specific biomarkers and understand the differential packaging and release of RNAs in various disease contexts, thereby facilitating biomarker discovery and therapeutic target identification.

#### Comparative profiles of pathway enrichment and tissue/cell origins in EVPs and cfRNAs

**Figure 4.**
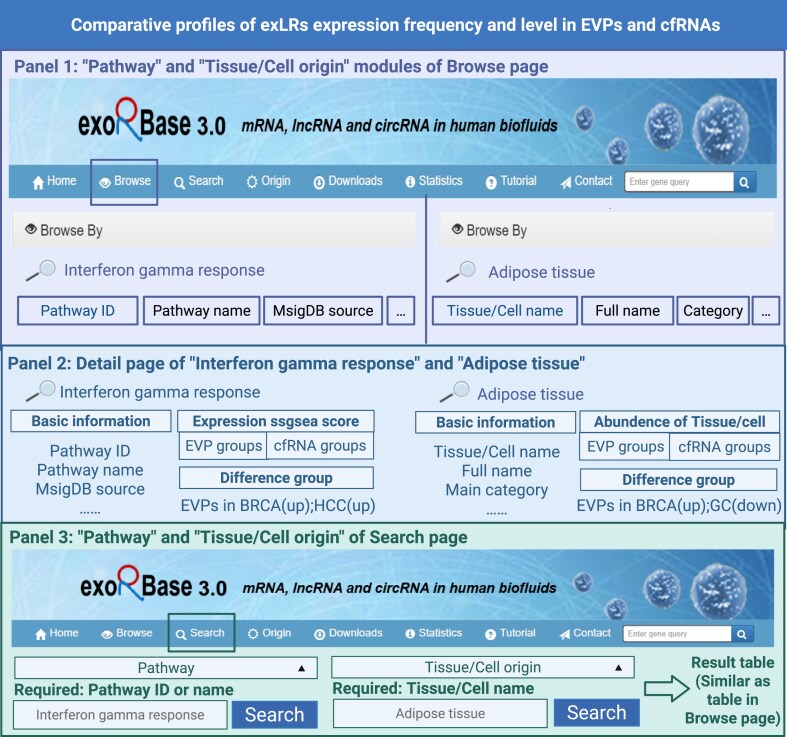
Comparative profiles of pathway enrichment and tissue/cell origin in EVPs and cfRNAs. Panel 1 describes the “Pathway” and “Tissue/Cell origin” modules of the Browse page showing search results for “Interferon gamma response” pathway (left) and “Adipose tissue” (right) with basic information fields displayed. Panel 2 describes the Detail pages of “Interferon gamma response” pathway (left) and “Adipose tissue” (right), displaying basic information, expression ssgsea scores or abundance across EVP and cfRNA groups, and differential expression patterns between groups. Panel 3 describes the “Pathway” and “Tissue/Cell origin” modules of the Search page where users can input pathway names or tissue/cell names to retrieve comprehensive information displayed in result tables.

Users can explore pathways and cellular or tissue origins of interest via the Browse and Search interfaces. For example, if “Interferon gamma response” is the pathway of interest, users can search for this pathway in both the Browse and Search pages. In the Browse interface (Fig. 4, Panel 1, left), the basic information for the pathway is displayed after searching. Clicking the blue link under “Pathway ID” navigates to the pathway Detail page (Fig. 4, Panel 2), where comprehensive details and statistics are provided, including basic information such as Pathway ID, Pathway name, and MsigDB source, expression ssgsea scores across EVPs and cfRNAs groups, and differential analysis results between various groups. The differential group analysis reveals that this pathway is upregulated in EVPs from BRCA patients compared to EVPs from healthy controls and upregulated in HCC compared to healthy controls. In the Search interface (Fig. 4, Panel 3, left), clicking the Search button displays basic pathway statistics similar to the Browse interface.

For tissue and cell origins, users can search for their tissue or cell type of interest, such as “Adipose tissue,” via the Browse and Search interfaces (Fig. 4, Panel 1, right and Panel 3, right). The display and navigation are consistent with the pathway module: searching displays the basic information, and clicking the blue link “Tissue/Cell name” leads to the Detail page (Fig. 4, Panel 2, right), which provides comprehensive tissue and cell abundance statistics across EVPs and cfRNAs groups. The differential group analysis shows that adipose tissue abundance is upregulated in EVPs from BRCA patients (compared to EVPs from healthy controls) and downregulated in GC (compared to healthy controls). This enables a direct comparison of pathway enrichment and tissue/cell origin of exLRs between EVPs and cfRNAs, revealing disease-specific biological features.

#### Direct comparison of exLR profiles in matched cancer samples between EVPs and cfRNAs

ExoRBase 3.0 includes matched cancer samples where both EVPs and cfRNAs are available under the same disease state. This enables users to directly compare the expression profiles of exLRs, pathways, or tissue/cell origins of interest between EVP and cfRNA states for the same cancer type. For example, users can investigate whether a target exhibits consistently high expression in both states, or whether there are significant differences, such as upregulation in one and downregulation in the other. Additionally, users can compare gene/pathway-level differences between EVP/cfRNA-derived samples by clicking “Compare,” as demonstrated in the tutorial (http://www.exorbase.org/tutorial.html). Such comparative analysis provides insights into the cancer-specific diagnostic potential and biological preferences of exLRs depending on their origin. Currently, the database supports matched EVP and cfRNA samples from GC, HCC, NSCLC, and CRC. Users can compare exLRs, pathways, or tissue/cell origins across these states to identify state-specific features. These comparisons can be performed in the “Compare” section of each exLR, pathway, or tissue/cell origin detail page, where differences between EVP and cfRNA datasets for a given cancer type are visually displayed.

### Enhanced visualization features in exoRBase 3.0

**Figure 5.**
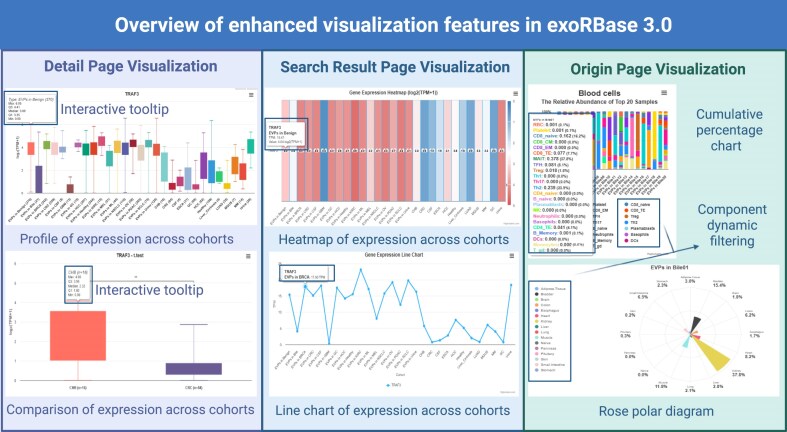
Overview of enhanced visualization features. The figure summarizes the major visualization upgrades implemented in exoRBase 3.0. The left panel shows interactive boxplots for profiling and comparing expression patterns across cohorts. The middle panel presents a dynamic heatmap and line chart for visualizing expression across cohorts. The right panel displays cumulative percentage charts and rose polar diagrams for analyzing tissue and cell origin distributions, with support for real-time component filtering.

In exoRBase 3.0, we have significantly upgraded our data visualization capabilities by implementing Highcharts for dynamic and interactive plotting across all main pages. Compared to previous static graphics, these enhancements provide users with faster rendering, richer interactivity, and a more intuitive exploration experience (Fig. 5). Key improvements include interactive boxplots for profiling and comparing expression patterns of exLRs, pathways, and tissue/cell origins in the Detail page; dynamic heatmaps and line charts for visualizing average expression differences across cohorts in the Search Result page; and rose polar diagrams and cumulative percentage charts for examining tissue and blood cell origin distributions in the Origin page. All visualizations now support mouseover tooltips for detailed data display, while flexible real-time component filtering is available for cumulative percentage charts and rose polar diagrams in the Origin page. These upgrades enable users to seamlessly explore and compare complex multi-cohort datasets, gaining deeper biological insights with greater ease.

## Discussion

Since its initial launch as a pioneering repository for exLRs derived from human blood exosomes, exoRBase has become an indispensable resource in liquid biopsy research, achieving remarkable community impact with over 600 citations and 130 000 global users (as of June 2025) [5, 6]. ExoRBase 3.0 represents a transformative advancement by providing the first comprehensive platform for integrated analysis of exLRs derived from both EVP and cfRNA fractions. This major update incorporates ∼3000 human biofluid samples—a three-fold increase over version 2.0—and expands transcriptomic coverage to include 126 816 circRNAs (versus 79 084 in v2.0), 15 961 lncRNAs, and 19 927 mRNAs across blood, urine, CSF, and bile. The platform’s enhanced analytical capabilities, including ssGSEA functional annotation across over 12 900 MSigDB pathways—a 112% increase from version 2.0—and refined cellular deconvolution algorithms for 16 tissue types and 23 blood cell subtypes, enable researchers to perform sophisticated comparative analyses between EVP- and cfRNA-derived exLRs while tracing their cellular origins.

The integration of EVP and cfRNA data addresses critical gaps in exRNA research driven by recent scientific advances. Following discoveries that RNA cargo can be transported by both membrane-bound vesicles and non-membranous particles such as exomeres and supermeres, exoRBase 3.0 adopts the latest consensus terminology “EVPs” to encompass this diversity of carriers [12–15]. Simultaneously, cfRNAs have emerged as clinically valuable biomarkers, with recent studies demonstrating their utility in pregnancy monitoring and lung cancer diagnosis [9–11]. These cfRNAs represent long transcripts (mRNAs or lncRNAs) that share molecular overlaps with EVP-derived long RNAs, necessitating their integration under the unified framework of exLRs. By incorporating ∼600 cfRNA datasets from seven different studies [29–35 and unifying EVP-derived and cell-free long RNAs, exoRBase 3.0 enables cross-modal biomarker discovery and supports hypothesis generation regarding the shared or distinct biological roles of RNA species across different extracellular compartments.

ExoRBase 3.0 offers unparalleled advantages over existing resources in the exRNA domain (Table 2). While databases such as exRNA Atlas focus on small RNA species and employ computational deconvolution methods and cfOmics catalogs cell-free multi-omics data, they lack comprehensive circRNA annotations and host fewer than 20 000 RNA entries [36, 37]. EV-focused repositories, including EVAtlas and Vesiclepedia 2024, face significant limitations: EVAtlas lacks cfRNA integration, Vesiclepedia focuses primarily on mRNA and miRNA across 56 organisms while excluding circRNA data [38, 39]. In contrast, exoRBase 3.0 consolidates multiple RNA species across diverse human biofluids while providing functional and cellular context through dynamic pathway-level visualization and comprehensive cell/tissue origin resolution.

**Table 2.** A comparison between exoRBase 3.0 and other databases

	exoRBase 3.0	exRNA Atlas	cfOmics	Vesiclepedia2024	EVAtlas
Total samples	2 913	11 033	11 345	3 481	2030
Sample types	Human EVP/cfRNA- derived long-RNA profiles from healthy and multiple diseases	Human and mouse exRNA samples (EVs and cfRNA)	Multi-omics liquid biopsy (cfDNA, cfRNA, proteome, metabolome) across 69 diseases	56 species; body fluids and cell culture supernatants (RNA, DNA, protein, lipid, metabolites)	Human EVs categorized by origin (tissue/cell lines) and disease states
Biofluid types	Bile, blood, CSF, urine	Bile, CSF, plasma, saliva, serum, urine; conditioned media	13 types (plasma, saliva, serum, urine, etc.)	Blood, urine, saliva, milk, ascites, CSF	Breast milk, plasma, saliva, serum, sperm, urine; primary cell, cell line
RNA biotypes	Long RNAs: mRNA, lncRNA, circRNA	Small RNAs: miRNA, tRNA, piRNA, snoRNA, snRNA	mRNA	mRNA, miRNA	NcRNAs: miRNA, snoRNA, piRNA, snRNA, rRNA, tRNA, YRNA
Functions	Expression browse/compare; pathway enrichment; EV-origin heterogeneity; visualization and downloads	Search by RNA; interactive visualization (PCA/UMAP, boxplots/heatmaps); downloads and API	Cross-omics browsing and visualization; IGV; correlation analyses; downloads	Open query and full database download; integrates with FunRich; accepts submissions	Browse/compare ncRNAs; filter by tissue/disease; specific expression; function/targets/drugs; method info; downloads

Several limitations warrant consideration in the current implementation. First, the representation of certain biofluid types, including saliva, ascites, and gastric fluid, remains limited, potentially constraining analyses across all clinically relevant contexts. Second, despite the rigorous application of cross-study normalization strategies, residual batch effects may persist and should be considered when integrating datasets from different studies or body fluids. Finally, the biological functions of many exLRs, particularly circRNAs, remain incompletely characterized and will require further experimental validation to fully realize their therapeutic potential.

Future developments will focus on addressing these limitations and expanding the platform’s capabilities. Planned enhancements include the incorporation of single-cell RNA sequencing and proteomic datasets to refine cell-type-specific signatures and explore RNA-protein co-expression networks. We will continue to expand biofluid representation and develop advanced computational methods to minimize batch effects across studies. Community participation through the platform’s data submission portal will be encouraged to continuously expand the resource’s scope and impact. These improvements will further establish exoRBase 3.0 as an essential tool for advancing exRNA research and accelerating the translation of exLR-based biomarkers into clinical practice.

## Data Availability

The exoRBase 3.0 database will be continuously maintained and updated. The database is now publicly accessible at http://www.exorbase.org/.
